# Bilateral giant femoropopliteal artery aneurysms: a case report

**DOI:** 10.1186/1752-1947-2-114

**Published:** 2008-04-20

**Authors:** Theodossios P Perdikides, Efthimios Avgerinos, Efstratios Christianakis, Theofanis Fotis, Anastasios Chronopoulos, Konstantinos X Siafakas, Nikolaos Pashalidis, Dimitrios K Filippou

**Affiliations:** 1 Department of Thoracic and Vascular Surgery, Hellenic Airforce Hospital, GR-11146 Galatsi, Athens, Greece

## Abstract

**Introduction:**

Popliteal artery aneurysms are the most common peripheral arterial aneurysms, and are frequently bilateral. Acute limb ischemia, rupture and compression phenomena can complicate these aneurysms when the diameter exceeds 2 cm.

**Case Presentation:**

We report an 82-year-old male patient with two giant femoropopliteal aneurysms, 10.5 and 8.5 cm diameters, managed in our institution. Both aneurysms were resected and a polytetrafluoroethylene (PTFE) femoropopliteal interposition graft was placed successfully. Management and literature review are discussed.

**Conclusion:**

We believe this is the first report in the medical literature of bilateral giant femoropopliteal aneurysms.

## Introduction

Popliteal artery aneurysms (PAAs) are defined as localized dilatations of the popliteal artery over 2 cm in diameter or more than 150% of the normal arterial calibre [1]. True PAAs are mostly atherosclerotic in origin.

Although they are the most common peripheral artery aneurysm, their prevalence in men aged 65 to 80 years is only 1% [2]. PAAs are often bilateral [3,4]. An associated abdominal aortic aneurysm (AAA) is present in approximately 50% of patients [3,4].

The most feared complication is the sudden development of acute ischemia caused by thrombosis of, or embolization from, the PAA. That is why it is often suggested that when a PAA has reached 2 cm in diameter, elective repair should be considered.

Here we report a patient with two giant femoropopliteal aneurysms managed successfully in our institution.

## Case presentation

An 82-year-old man who was a heavy smoker was referred to our hospital for the evaluation of bilateral huge femoropopliteal masses extending from the medial middle of the thigh to the knee. The patient complained of discomfort and bilateral impeded ambulation but no other particular symptoms were reported.

His past medical history included coronary artery disease being treated with medication, a known thoraco-abdominal aneurysm, prior abdominal aortic aneurysm repair and a right nephrectomy due to kidney donation for transplant to his daughter.

Physical examination revealed bilateral non-tender pulsatile masses. Both limbs had pedal pulses. His right knee was partially contracted.

**Figure 1:**
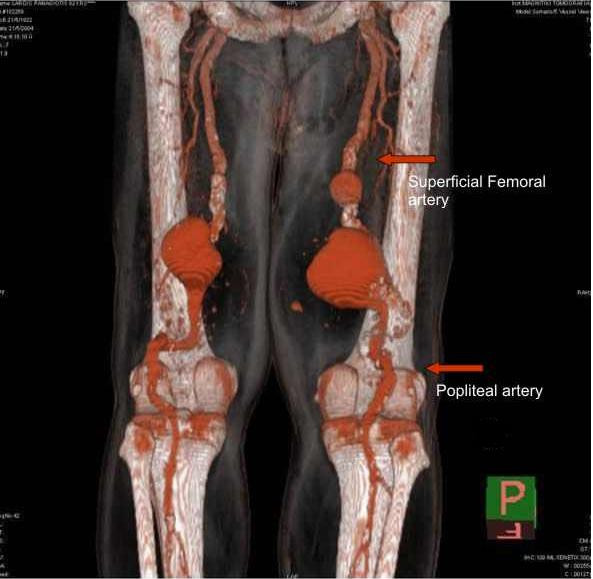
**CT angiography results.** A, B: 3D CT angiograms indicating the functional lumen of the two femoropopliteal aneurysms. C: CT scan indicating the maximum diameters of the popliteal aneurysms (10.5 cm on the right, 8.5 cm on the left).

The diagnosis of femoropopliteal aneurysms was suspected. Diagnostic assessment included multi-slice spiral computed tomography (CT) angiography which revealed two huge femoropopliteal aneurysms. The right one had a maximum diameter of 10.5 cm and the left a maximum diameter of 8.5 cm (Figure 1).

**Figure 2:**
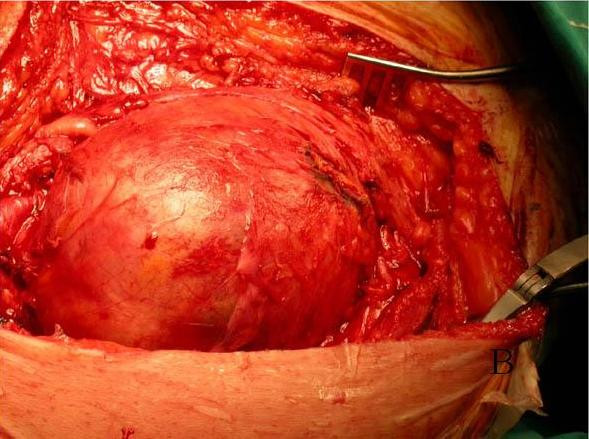
**Operative field**. A: Right femoropopliteal aneurysm. B: Left femoropopliteal aneurysm.

**Figure 3:**
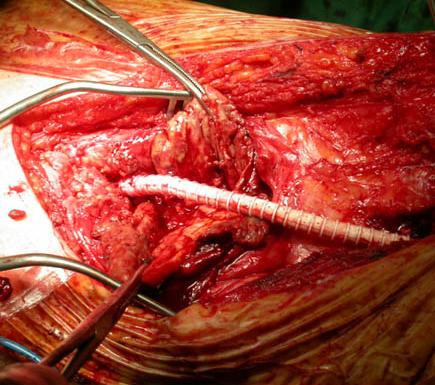
**Right aneurysm repair**. A: Interposition graft. B: Excised aneurysm.

Both aneurysms were resected following the same procedure, although there was a two-month interval between each resection. A 'classic' medial approach was used. The aneurysm was dissected carefully, incised longitudinally, the thrombi were evacuated, collaterals were oversewn from within the aneurysm, which finally was excised and replaced by an 8 mm PTFE femoropopliteal interposition graft (Figures 2 and 3). On both occasions, the postoperative course was uneventful. Two years later, arteriographic and Doppler examination showed patent bypass bilaterally.

## Discussion

Vascular surgical evaluation is essential for the differential diagnosis of thigh and popliteal masses, regardless of size.

Several reports indicate the relatively common incidence of PAAs, and also their bilateral nature. However, giant bilateral femoropopliteal aneurysms such as those presented here are a rarity, and are reported here for the first time in the literature.

PAAs can be a threat to the lower limbs, because of thromboembolic phenomena, occasional rupture and compression of adjacent structures. However, even elective repair of asymptomatic PAAs carries a relative risk, with a reported 1% of patients being left with residual symptoms [5]. The diameter of the PAA seems to relate to the symptoms; 2 cm PAAs are usually asymptomatic, while 3 cm aneurysms appear frequently with limb threatening ischemia [6].

Excision and/or decompression is the mainstay of the therapy for large popliteal aneurysms, with the primary goal being maintenance of foot viability. Secondary goals should be directed towards the alleviation of associated compressive features of the adjacent nerve and popliteal vein, which can cause neuropathies, or venous thrombosis [4,7,8]. In addition, reduction of the mass, in the case of very large lesions, to enable successful ambulation is a consideration.

Two standard operative approaches are optimal for popliteal aneurysm repair: medial and posterior. The usual method of dealing with large PAAs, as adopted in our case, is aneurysm excision and graft interposition through a medial exposure. Proximal and distal ligation combined with either popliteal-popliteal bypass or femoropopliteal bypass using a vein or synthetic graft is not recommended for large aneurysms because of the risk of aneurysm expansion through collaterals. Posterior exposures could facilitate sac decompression while visualizing adjacent neurovascular structures. The posterior approach lends itself to an endoaneurysmorrhaphy reconstructive technique, especially in the case of an above-to-below-knee popliteal artery bypass.

## Conclusion

Although surgical repair is associated with excellent long-term durability [9], during the last decade there has been increasing interest in using endovascular methods to treat PAAs. Patient selection for endovascular repair depends on suitable popliteal artery anatomy, the extent of aneurysmal degeneration, and the quality of tibial arterial runoff. The endovascular experience in giant PAAs is still limited, thus we considered surgery the optimal strategy for our patient.

## Competing interests

The authors declare that they have no competing interests.

## Authors' contributions

TPP, EA, EC, TF, AC, KXS and DKF were the attending physicians as well as the coordinating doctors (microbiologist, radiologist) responsible for the diagnosis and treatment of the patients and who provided the information. TPP and KXS helped to draft the manuscript. EC, TF, AC, NP and DKF wrote the final manuscript. EA, NP and DKF made the final revisions to the paper. All authors read and approved the final manuscript.

## Consent

Written informed consent was obtained from the patient for publication of this case report and accompanying images. A copy of the written consent is available for review by the Editor-in-Chief of this journal.
